# A Hierarchical Low-Rank Denoising Model for Remote Sensing Images Based on Deep Unfolding

**DOI:** 10.3390/s24144574

**Published:** 2024-07-15

**Authors:** Fanqi Shao, Xiaolin Feng, Sirui Tian, Tianyi Zhang

**Affiliations:** 1 China Electronic Technology Group Corporation, Beijing 100846, China; sfanq@163.com; 2 School of Electronic and Optical Engineering, Nanjing University of Science and Technology, Nanjing 210094, China; fengxiaolin@njust.edu.cn (X.F.); tiansirui@njust.edu.cn (S.T.)

**Keywords:** low-rank, hierarchical model, remote sensing image denoising, deep unfolding, edge preservation

## Abstract

Recently, the low-rank representation (LRR) model has been widely used in the field of remote sensing image denoising due to its excellent noise suppression capability. However, those low-rank-based methods always discard important edge details as residuals, leading to a common issue of blurred edges in denoised results. To address this problem, we take a new look at low-rank residuals and try to extract edge information from them. Therefore, a hierarchical denoising framework was combined with a low-rank model to extract edge information from low-rank residuals within the edge subspace. A prior knowledge matrix was designed to enable the model to learn necessary structural information rather than noise. Also, such traditional model-driven approaches require multiple iterations, and the solutions may be very complex and computationally intensive. To further enhance the noise suppression performance and computing efficiency, a hierarchical low-rank denoising model based on deep unrolling (HLR-DUR) was proposed, integrating deep neural networks into the hierarchical low-rank denoising framework to expand the information capture and representation capabilities of the proposed shallow model. Sufficient experiments on optical images, hyperspectral images (HSI), and synthetic aperture radar (SAR) images showed that HLR-DUR achieved state-of-the-art (SOTA) denoising results.

## 1. Introduction

Remote sensing data acquisition often relies on sensors mounted on satellites or aircraft, which can capture electromagnetic spectra in different bands, including visible light, infrared, microwaves, etc. Due to its capability to cover vast geographical areas and provide continuous observations, remote sensing technology has important applications in numerous fields such as agriculture, forestry, geology, meteorology, environmental protection, and urban planning [1]. However, remote sensing images are inevitably affected by various factors during acquisition and transmission, leading to the presence of noise in the images [2].

In recent years, the rapid development of deep learning has injected new vitality into various fields [3,4], particularly in the domain of image processing [5,6,7]. Utilizing the powerful learning and representational capabilities of deep neural networks can more effectively solve many challenges and problems in image processing [8], which also brings inspiration to the issue of remote sensing image denoising. Compared to traditional image processing methods, deep learning, through an end-to-end training process, can automatically learn high-level, semantically rich feature representations from a large amount of data. These learned features not only have better robustness and generalization capability for image-denoising tasks but also provide richer image descriptions and understanding.

Deep learning methods have been proven to have significant effects in the task of denoising remote sensing images. For optical remote sensing images, a method of remote sensing image denoising based on a Generative Adversarial Network (GAN) named “Restoration Generative Adversarial Network with ResNet and DenseNet” (RRDGAN) was proposed [9]. This method acquires better-quality images by incorporating denoising and SR into a unified framework. Huang et al. integrated deep convolutional neural network (DCNN) denoiser prior into a unidirectional variation (UV) model, named UV-DCNN, to simultaneously destripe and denoise optical remote sensing images [10]. For hyperspectral images (HSI) images, Xie et al. introduced the Deep Spatio-Spectral Bayesian Posterior Network (DSSBPNet) for hyperspectral images [11]. DSSBPNet combines Bayesian variational posterior with deep neural networks, where a deep spatial-spectral network divides the input image into three parts, generating spatial-spectral gradients for each. Different convolutions are used in different parts of the DSS network. Meanwhile, noise estimation of the original data, noise distribution, and sparse noise gradients constitute the Bayesian posterior approach. Finally, the forward-backward propagation method is used to connect DSS with the Bayesian posterior. A novel, deep-learning framework for 3-D HSI denoising [12] was proposed, which decomposes 3-D filtering into 2-D spatial filtering and 1-D spectral filtering. This method can achieve substantial savings on the number of network parameters to keep the computational complexity low. For synthetic aperture radar (SAR) images, a novel two-component deep learning (DL) network was proposed for SAR despeckling [13]. First, the texture estimation subnetwork is constructed to produce the texture level map (TLM), which evaluates the randomness and scale of the texture distribution. Then, the noise removal subnetwork learns a spatially variable mapping between the noise and clean images with the help of TLM.

However, we cannot ignore the important role of traditional models in the field of denoising. Low-rank representation (LRR) models have been widely explored and improved in the field of remote sensing image denoising [14,15]. A method called NAILRMA [16] proposed an iterative regularization framework aiming to achieve denoising of signal subspaces. In NAILRMA, an adaptive iteration factor selection method based on noise variance was applied to each band of the hyperspectral image (HSI). Additionally, some tensor-based low-rank denoising methods [17,18,19] have also demonstrated satisfactory results. The Fisher–Tippett distribution-based WNNM (FT-WNNM) [20] attempts to recover underlying low-rank components from patch group matrices. The Composite Regularization Method for Spot Noise Reduction (CRM-SR) [21] introduces regularization terms, including TV regularization and NLLR regularization. However, determining the regularization parameters involved in the CRM-SR model is challenging. The NLLR framework [22] decomposes speckle noise into low-rank components with proposed truncation and weighted nuclear norm regularization. Considering the effectiveness of the LRR model, more and more denoising algorithms have combined low-rank models with deep learning denoising methods. 

Zhang et al. proposed a Deep Low-Rank Prior (DLRP) [23] for remote sensing image denoising, firstly using the low-rank characteristics of nearby non-local self-similar blocks arranged in dictionary order to model the Global Objective Function (GOF). Secondly, with the help of an alternating iterative strategy, GOF can be easily decomposed into two independent sub-problems. Among them, the low-rank minimization denoising problem can be solved through learning with a deep convolutional neural network. Nguyen et al. proposed an HSI denoising method using deep image prior with sparse and low-rank prior [24]. However, hyperparameter selection in this method is still an open question. Sun et al. proposed a HSI denoising via LRR and CNN denoiser [25]. The sparse-based low-rank representation can explore the global correlations in both the spatial and spectral domains, and the CNN-based denoiser can represent the deep prior, which cannot be designed by traditional restoration models. However, such denoising methods often have a common problem, that is, they will filter out significant edge signals as noise, which will degrade the quality of images. Zhang et al. developed a self-supervised HSI denoising method via integrating a model-driven strategy with a data-driven strategy [26]. The proposed framework simultaneously cooperates with the spectral low-rankness prior and deep spatial prior (SLRP-DSP) for HSI self-supervised denoising. Regarding the characteristics of SAR images, more novel deep networks were proposed. Xiong et al. decomposed the SAR image in the SAR imaging-despeckling observation model into a sparse matrix and a low-rank matrix, and then established an optimization problem with the corresponding sparse and low-rank priors [27]. A despeckling model was proposed that uses deeper convolutional neural networks [28], which was never used before, as far as authors are concerned, for diminishing speckles in noisy SAR images. Multiple skip connections from the ResNet model are also employed in the authors’ proposed architecture. However, these denoising methods often do not take into account the issue of edge preservation in the denoising results, leading to the loss of some important structural information in the denoised images.

In this paper, we combine the shallow low-rank denoising model with an autoencoder, following the method in [29], to construct a two-level deep encoder. By applying deep unfolding, the shallow LRR denoising model was introduced into the realm of deep learning. Thus, a hierarchical low-rank denoising model based on deep unrolling (HLR-DUR) was proposed. Integrating the characteristics of autoencoders, a two-level deep autoencoder was established to optimize the iterative solution process for the low-rank and edge components of remote sensing images. Utilizing the advantages of deep neural networks, the model captures more complex patterns in the data, thereby enhancing the effectiveness of image denoising. Finally, experiments conducted on optical remote sensing images, hyperspectral images, and SAR images demonstrated that HLR-DUR significantly outperforms shallow models across various metrics.

The main contributions can be summarized as follows:

1. We think the residuals of traditional low-rank models contain not only noise but also high-frequency edge information that has been filtered out. Therefore, we tried to re-extract edge information from the residuals to construct a hierarchical low-rank model for denoising remote sensing images. To better achieve edge extraction, we introduced manifold learning into the model, ensuring that useful structural information, rather than noise, is extracted in the edge subspace. Additionally, a new prior knowledge regulation was designed to distinguish between clean pixels and noisy pixels, guiding the model to learn useful information during the denoising process;
2. Due to the number of iterations required, traditional LRR models usually take a long time to process large-sized remote sensing images. To tackle this problem, we designed a hierarchical denoising model based on deep unfolding by combining shallow models with deep autoencoders. By mapping traditional iterative optimization algorithms onto the structure of neural networks, each iterative step was transformed into a layer of the network. This technique enhances the interpretability of neural networks, making each layer no longer a black box model but rather corresponding to a specific optimization step;
3. Experiments conducted on three types of images—optical remote sensing images, hyperspectral images, and SAR images—demonstrated the effectiveness of our proposed HLR-DUR model. HLR-DUR not only enhances denoising performance but also better preserves sufficient edge detail compared to existing methods, achieving better edge-preserving denoising.

## 2. Related Work

Wang et al. proposed that a low-rank prior and a fully convolutional Auto Encoder (AE) can be incorporated through modeling an energy minimization problem [30], which is similar to the Robust Principal Component Analysis (RPCA) model. 

Given a data matrix **X**, it can be decomposed as [31]:

(1) X=Z+E

where Z represents the low-rank part and E is a sparse matrix, which represents residual. Z and E can then be optimized by solving the following energy minimization problem:

(2) $$\begin{array}{l} \underset{Z,E}{\min}{\left\|Z\right\|}_{*}+\lambda {\left\|E\right\|}_{1}\\ s.t.X=Z+E \end{array}$$

where ${\left\|\cdot \right\|}_{*}$ denotes nuclear norm, ${\left\|\cdot \right\|}_{1}$ denotes $l_{1}$ norm, and λ is the regularization parameter.

Based on this model, the low-rank prior and a fully convolutional AE are incorporated by modeling an energy minimization problem, which can be mathematically depicted as follows:

(3) $$\begin{array}{l} \underset{Z,\theta }{\min}\frac{1}{2}{\left\|(f_{\theta }(Y)-X)W\right\|}^{2}+\lambda {\left\|Z\right\|}_{*}\\ s.t.Z=f_{\theta }(Y) \end{array}$$

where **Y** has the same dimensionality as the hyperspectral image, $f_{\theta }(Y)$ is the network which uses the uniform distribution as a prior to generate a reconstruction of the background, and **W** is the adaptive-weighted map.

Then, the problem can be solved by the Alternating Direction Method of Multipliers (ADMM). The model can be described as

(4) $$\underset{Z,\theta }{\min}\frac{1}{2}{\left\|(f_{\theta }(Y)-X)W\right\|}^{2}+\lambda {\left\|Z\right\|}_{*}+\frac{\mu }{2}{\left\|Z-f_{\theta }(Y)\right\|}^{2}+S^{T}(Z-f_{\theta }(Y))$$

where **S** stands for the Lagrange multiplier, and μ is a penalty parameter.

Finally, the above-shown optimization problem can be split into three subproblems, and each subproblem can be solved alternately in an iterative procedure.

## 3. Proposed Methodology

As previously mentioned, the presence of noisy data can destroy the low-rank structure of the data. So, the LRR model splits the data into clean parts and noisy parts, separating the noisy data while maintaining the low-rank structure of the data. However, the LRR model has a drawback that cannot be ignored. Specifically, it strives to maintain a low-rank structure by filtering out useful high-frequency information, which then causes important image edges to be discarded as part of residuals. Motivated by this phenomenon, we first try to construct a hierarchical low-rank model to extract edge details from residuals. Then, we try to combine the proposed hierarchical model with deep neural networks to further improve the denoising effect.

### 3.1. Hierarchical Low-Rank Model

#### 3.1.1. Model Formulation

To address the issue of traditional low-rank models failing to preserve structural edge information, we modify the traditional low-rank model using a prior knowledge matrix. This modification allows the model to better distinguish between clean and noisy pixels. The model can be represented as

(5) $$\begin{array}{l} \underset{Z,E}{\min}{\left\|Z\right\|}_{*}+\lambda {\left\|E\right\|}_{2,1}\\ s.t.\;H\circ X=H\circ Z+E \end{array}$$

where X represents the original noisy image, Z represents the low-rank component, and E represents the low-rank residual. H is the prior knowledge matrix used in this model, “◦” denotes the Hadamard product, and λ is an adjustable parameter used to balance the residual term.

It is crucial to extract the edge information that has been filtered out from the residuals. Building on (5), we innovatively introduce a constraint term $H\circ E=H\circ E_{1}+H\circ E_{2}$, where $E_{1}$ represents the edge part extracted from the residual, and $E_{2}$ represents the noise component in the residual. By further decomposing the residual, the filtered edge information can be obtained, thereby enhancing the quality of the denoising results. However, considering the different requirements for extracting residuals and low-rank components, we further set up hierarchical prior knowledge information, implemented through different coefficients, to meet different requirements. Specifically, when extracting the low-rank part, the goal is to achieve as clean denoising results as possible, that is, the less noise, the better, even if it means that the low-rank part of the image may have blurred edges and lose some detail. When extracting edge structures, the focus is more on effectively extracting rich edge information, even if some noise is introduced in the process of extracting edges. To achieve this goal, we further propose a hierarchical prior knowledge to better extract the edge details from the residuals, with the model presented as follows:

(6) $$\begin{array}{l} \underset{Z,E,E_{1},E_{2}}{\min}{\left\|Z\right\|}_{*}+\lambda {\left\|E\right\|}_{2,1}\\ s.t.\;H_{1}\circ X=H_{1}\circ Z+E\\ \;\;\;H_{2}\circ E=H_{2}\circ E_{1}+E_{2} \end{array}$$

It is noteworthy that in the constraints of (6), H matrices with different subscripts appear, as a special design of the hierarchical model. Here, $H_{1}$ is used to obtain the clean low-rank part from the noisy image, while $H_{2}$ is used to find more edge details from the residuals. As mentioned above, $H_{1}$ should be more stringent to ensure that as much noise as possible is eliminated. In contrast, the constraints on $H_{2}$ are relatively lenient because $H_{2}$ needs to extract more useful information from the residuals.

However, extracting sufficient edge information from residuals containing a large amount of noise is not an easy task. To ensure that our model learns the required edge information rather than noise, and to ensure that good data geometric structures are captured within the model, a manifold learning framework was also introduced to the proposed model [32]. Manifold learning is a machine learning technique that explores and exploits the underlying low-dimensional structures of high-dimensional data, based on the idea that while data may be presented in high-dimensional spaces, they actually distribute along some low-dimensional manifolds. The purpose of manifold learning is to reveal these low-dimensional structures to better understand the essential characteristics of the data. By introducing manifold learning, robust subspace projections $V=(v_{1},v_{2},\ldots ,v_{r})$ can be learned from the residuals $E_{1}$, allowing for the extraction of edge subspaces. By introducing dynamic affinity graph regularization, manifold learning can be incorporated into the original model, with the formula represented as follows:

(7) $$\begin{array}{l} \underset{V,Z,W,E,E_{1},E_{2}}{\min}\frac{1}{2}\sum_{i,j}{\left\|V^{T}e_{1,i}-V^{T}e_{1,j}\right\|}_{2}^{2}W_{ij}+\lambda {\left\|W\right\|}_{F}^{2}\\ s.t.\;H_{1}\circ X=H_{1}\circ Z+E\\ \;\;\;H_{2}\circ E=H_{2}\circ E_{1}+E_{2}\\ \;\;\;V^{T}V=I\\ \;\;\;W1=1,W_{ii}=0,W\geq 0 \end{array}$$

where $e_{1,i}$ and $e_{1,j}$ represent the values of corresponding samples in the residual $E_{1}$, and $1/2\sum_{i,j}{\left\|V^{T}e_{1,i}-V^{T}e_{1,j}\right\|}_{2}^{2}$ is an increased coupling term. It is a part of our model because we do not know which part of the data is affected by noise.W is the dynamic affinity graph matrix, and λ is an adjustable parameter used to balance the dynamic affinity graph. 

For two similar samples $x_{i}$ and $x_{j}$, if the values of corresponding $e_{1,i}$ and $e_{1,j}$ are very small, this also suggests that the distance between them will be very small. Therefore, it can be considered that they primarily contain edge information, which are the edge details to be extracted from the residual E. Conversely, if they are part of the noise, the distance between them will be increased. Based on this assumption, a weighting coefficient $W_{ij}$ is introduced to adaptively learn projections V with similar residuals. When two residual samples are similar, $W_{ij}$ is set to non-zero. Otherwise, $W_{ij}$ is set to zero to mitigate the impact of noise. At the same time, three non-negative constraints $W_{ii}=0$, W≥0, and W1=1 are also added to the model, and these three constraints are used to ensure that the affinity graph will be normalized. Finally, the complete model is as follows:

(8) $$\begin{array}{l} \underset{V,Z,W,E,E_{1},E_{2}}{\min}\frac{1}{2}\sum_{i,j}{\left\|V^{T}e_{1,i}-V^{T}e_{1,j}\right\|}_{2}^{2}W_{ij}+\lambda_{1}{\left\|Z\right\|}_{*}+\lambda_{2}{\left\|E\right\|}_{2,1}+\lambda_{3}{\left\|W\right\|}_{F}^{2}\\ s.t.\;H_{1}\circ X=H_{1}\circ Z+E\\ \;\;\;H_{2}\circ E=H_{2}\circ E_{1}+E_{2}\\ \;\;\;V^{T}V=I\\ \;\;\;W1=1,W_{ii}=0,W\geq 0 \end{array}$$

where $\lambda_{1}$, $\lambda_{2}$, $\lambda_{3}$ are adjustable parameters used to balance the different terms.

To solve (8), we employed the Augmented Lagrange Multiplier method (ALM). The coupling term $1/2\sum_{i,j}{\left\|V^{T}e_{1,i}-V^{T}e_{1,j}\right\|}_{2}^{2}$ can be rewritten as $tr(V^{T}(FL{\hat{E}}^{T})V)$, where L=D−W is the graph Laplacian operator of W, and $D=diag\sum_{i}W_{ij}$ is the diagonal matrix. Then, a relaxation variable S to denote Z, a relaxation variable $E_{1}$ to denote $\hat{E}$ and a relaxation variable R to denote E are introduced to make (8) easier to solve. Additionally, another variable F is introduced to avoid the Sylvester equation during the solution process for E. Therefore, (8) is reformulated as

(9) $$\begin{array}{l} \underset{V,Z,W,E,E_{1},E_{2}}{\min}tr(V^{T}(FL{\hat{E}}^{T})V)+\lambda_{1}{\left\|Z\right\|}_{*}+\lambda_{2}{\left\|E\right\|}_{2,1}+\lambda_{3}{\left\|W\right\|}_{F}^{2}\\ s.t.\;H_{1}\circ X=H_{1}\circ Z+E\\ \;\;\;H_{2}\circ E=H_{2}\circ E_{1}+E_{2}\\ \;\;\;V^{T}V=I\\ \;\;\;W1=1,W_{ii}=0,W\geq 0\\ \;\;\;S=Z,E_{1}=\hat{E},F=\hat{E},R=E \end{array}$$

#### 3.1.2. Prior Knowledge Regulation Construction

Firstly, we constructed the basic prior knowledge matrix based on the work of ROLD [33]. ROLD is a rank-ordered logarithmic difference, which can identify more noisy pixels with fewer false hits. With such statistics as cornerstones, we tried to construct our prior knowledge matrix. The uncorrupted probability $h_{i}$ of each pixel $k_{i}$ using ROLD is as follows:

(10) $$h_{i}=\exp(-\alpha ROLD(k_{i}))$$

where α is the coefficient used to obtain image features under different requirements. When α is large, the difference between uncontaminated pixels and contaminated pixels will be widened, and the final result will filter most speckle noise, but more details such as edges will also be filtered out together. On the contrary, when we set α small, it is more likely to retain the edge details of images even though some speckle noise may also be left on images.

Furthermore, we adapted the abovementioned prior knowledge matrix to make it more compatible with SAR images. The backscattered signatures from point targets are dominated by a small number of strong scatterers, which will make them classified as noisy pixels. Therefore, we adopted the method of the Enhanecd Lee filter (EnLee) [34] to divide all pixels into three categories, and combined it with (10) to make the prior knowledge matrix more suitable for SAR images. Through using the hierarchical prior knowledge matrices, our method can better learn the intrinsics of the low-rank part and edge part, and obtain clearer results.

Usually, two classes are considered in traditional filters: homogeneous and heterogeneous. Enhanced Lee filter presented that isolated point targets need special treatment as a third class. For an NLOOK intensity image, it is filtered as follows:

(11) $$\hat{R}(t)=\left\{ \begin{array}{l} \bar{I},C_{i}<C_{\min}\\ \bar{I}(t)+\omega (t)(I(t)-\bar{I}(t))\\ I,C_{i}>C_{\max} \end{array}\right.,C_{\min}\leq C_{i}\leq C_{\max}$$

where $C_{i}=\frac{std}{\bar{I}}$, std is the standard deviation within the filter window. $C_{\min}=\frac{1}{\sqrt{NLOOK}}$, $C_{\max}=\sqrt{1+\frac{2}{NLOOK}}$.

The weight function can be written as

(12) $$\omega (t)=\exp(\frac{-(C_{i}-C_{\min})}{\left(C_{\max}-C_{i}\right)})$$

Learning from EnLee, we divide all pixels into three categories to obtain the weight matrix ε:

(1) $C_{i}>C_{\max}$, which means that this pixel is a point target and needs to be preserved. So, we set the corresponding value in ε to 0.9 (a very big value).
(2) $C_{\min}<C_{i}<C_{\max}$, update ε as 1−ω according to (12).
(3) $C_{i}<C_{\min}$, which represents that this pixel is a noisy pixel and needs to be smoothed. So, we set the corresponding value in ω to 0.1 (a respectively small value).

Then, the new prior knowledge matrix is constructed as follows:

(13) $$H_{i}=h_{i}\circ \varepsilon$$

Finally, $H_{i}$ needs to be normalized to represent the probability of 0–1.

### 3.2. Deep Unfolding Model HLR-DUR Based on Autoencoder

In recent years, deep low-rank representation models have received increasing attention as a potential solution for designing interpretable neural networks. By mapping traditional iterative optimization algorithms into the structure of neural networks, each iterative step is transformed into a layer of the network, enhancing the interpretability of neural networks. Each layer of the network is no longer a black-box model but corresponds to a specific optimization step. In the unfolded network, models based on traditional physical meanings are used as the basic architecture, and the parameters that need manual adjustment are transformed into trainable parameters of the network. The core idea of the deep unfolding technique is to utilize deep neural networks to simulate and optimize the iterative process of low-rank representation. Therefore, based on (9), the new model can be described as

(14) $$\begin{array}{l} \underset{V,Z,S,W,E,E_{1},E_{2},\hat{E},F,R}{\min}tr(V^{T}(FL{\hat{E}}^{T})V)+\lambda_{1}{\left\|S\right\|}_{*}+\gamma_{1}{\left\|Z-f_{de1}(X;\theta_{1})\right\|}_{F}^{2}\\ +\lambda_{2}{\left\|E\right\|}_{2,1}+\gamma_{2}{\left\|E_{1}-f_{de2}(E;\theta_{2})\right\|}_{F}^{2}+\lambda_{3}{\left\|W\right\|}_{F}^{2}\\ s.t.\;H_{1}\circ X=H_{1}\circ Z+E,H_{2}\circ E=H_{2}\circ E_{1}+E_{2}\\ \;\;\;E_{1}=\hat{E},S=Z,R=E\\ \;\;\;V^{T}V=I,W1=1,W_{ii}=0,W\geq 0 \end{array}$$

where $f_{de1}(X;\theta_{1})=g(W^{L}\ldots g(W^{i}\ldots g(W^{2}X)))$ denotes a multi-layer deep autoencoder, $\theta_{1}=\{W^{2},\ldots ,W^{L}\}$ is a set of learning parameters used for learning the low-rank component. Additionally, $f_{de2}(E;\theta_{2})$ is used to extract edge part, and $\theta_{2}$ is also a set of learning parameters.

Before solving for the optimal solution of each variable in (14), it is necessary to first obtain its Augmented Lagrangian Multiplier (ALM) function; the complete equation is as follows:

(15) $$\begin{array}{l} \underset{V,Z,S,W,E,\hat{E},E_{1},E_{2},F,R}{\min}tr(V^{T}(FL{\hat{E}}^{T})V)+\lambda_{1}{\left\|S\right\|}_{*}+\lambda_{2}{\left\|E\right\|}_{2,1}+\lambda_{3}{\left\|W\right\|}_{F}^{2}\\ \;\;\;\;\;\;+\gamma_{1}{\left\|Z-f_{de1}(X;\theta_{1})\right\|}_{F}^{2}+\gamma_{2}{\left\|E_{1}-f_{de2}(E;\theta_{2})\right\|}_{F}^{2}\\ \;\;\;\;\;\;+tr(Y_{1}^{T}(H_{1}\circ X-H_{1}\circ Z-E))+tr(Y_{2}^{T}(H_{2}\circ R-H_{2}\circ E_{1}-E_{2}))\\ \;\;\;\;\;\;+tr(Y_{3}^{T}(S-Z))+tr(Y_{4}^{T}(\hat{E}-E_{1}))+tr(Y_{5}^{T}(F-\hat{E}))+tr(Y_{6}^{T}(R-E))\\ \;\;\;\;\;\;+\frac{\mu }{2}({\left\|H_{1}\circ X-H_{1}\circ Z-E\right\|}_{F}^{2}+{\left\|H_{2}\circ R-H_{2}\circ E_{1}-E_{2}\right\|}_{F}^{2}\\ \;\;\;\;\;\;+{\left\|S-Z\right\|}_{F}^{2}+{\left\|\hat{E}-E_{1}\right\|}_{F}^{2}+{\left\|F-\hat{E}\right\|}_{F}^{2}+{\left\|R-E\right\|}_{F}^{2})\\ s.t.\;V^{T}V=I\\ \;\;\;W1=1,W_{ii}=0,W\geq 0 \end{array}$$

where $Y_{1}$, $Y_{2}$, $Y_{3}$, $Y_{4}$, $Y_{5}$ and $Y_{6}$ are the introduced Lagrange multiplier.

By establishing a two-level deep encoder, the traditional hierarchical low-rank model is extended to a deep learning network. In traditional iterative algorithms, the denoising task is typically addressed through multiple iterative steps, with each step being computed based on the output of the previous one. In deep unfolding, these iterative steps are “unfurled” into a sequence of layers, each layer corresponding to an iterative step. Within these unfolded layers, the iterative process of low-rank representation is simulated and optimized through a deep autoencoder network.

**Figure 1:**
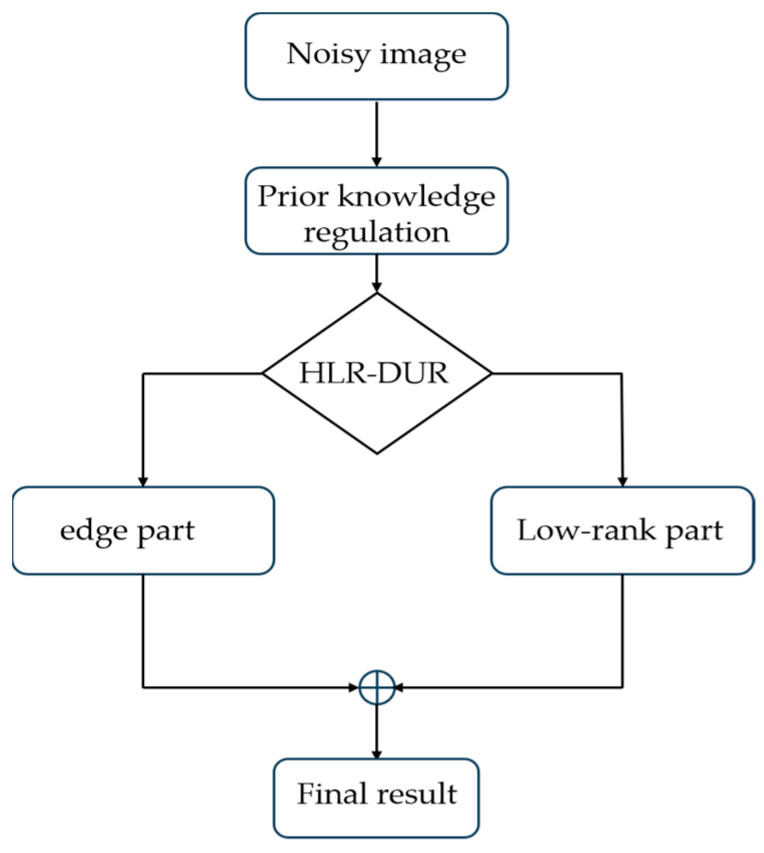
The flowchart of the proposed method.

The flowchart of the proposed model is depicted in Figure 1. Initially, the noisy image undergoes preprocessing with a prior knowledge matrix. Subsequently, the preprocessed noisy image is fed into HLR-DUR to obtain the low-rank and edge components. Finally, the denoised edge and low-rank components are merged to produce a clean image.

**Figure 2:**
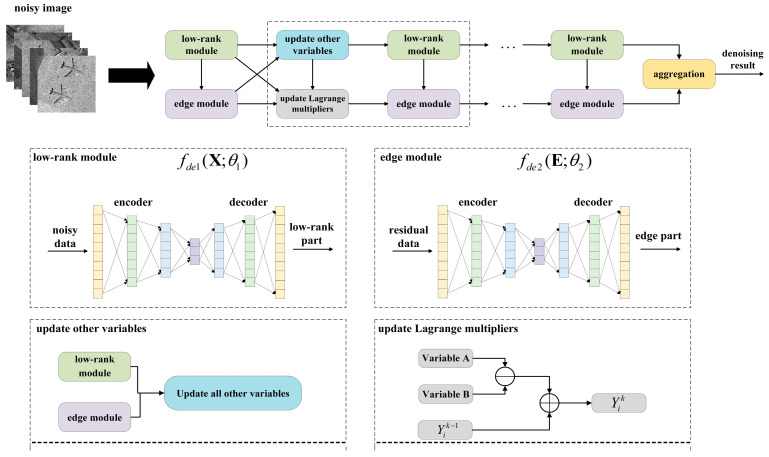
The structure of the HLR-DUR model.

The above iterative process is unfolded into a neural network, whose general architecture and components are shown in Figure 2. It consists of four main modules: the low-rank module, the edge module, the update module for the other parameters, and the Lagrangian multiplier module. Each module corresponds to the subproblem.

Next, we introduce each module.

- low-rank module

The low-rank module is used to compute the clean low-rank component extracted from noisy data. $\theta_{1}$ is computed by minimizing $L_{\theta 1}$ with respect to the weight parameters $\theta_{1}$ while keeping other parameters fixed. The optimal solution is obtained by solving the following subproblem:

(16) $$L_{\theta 1}={\left\|Z-f_{de1}(X;\theta_{1})\right\|}_{F}^{2}$$

The optimization is performed using the Gradient Descent (GD) algorithm, and the formula can be written as follows:

(17) $$\theta_{1}=\theta_{1}-\zeta \partial L_{\theta 1}/\partial \theta_{1}$$

where ζ is the learning rate and $\partial L_{\theta 1}/\partial \theta_{1}$ represents the gradient of $L_{\theta 1}$.

- edge module

The edge module is used to extract useful structural edge information from the residual part. Its solution process is similar to that of the low-rank module, which can be described as

(18) $$L_{\theta 2}={\left\|E_{1}-f_{de2}(E;\theta_{2})\right\|}_{F}^{2}$$

(19) $$\theta_{2}=\theta_{2}-\zeta \partial L_{\theta 2}/\partial \theta_{2}$$

- other variables

This module is used to update other variables apart from the low-rank part and the edge part. We have eight variables that need to be updated: $V,S,W,E,\hat{E},E_{2},F,R$. We adopt the alternating direction method of multipliers (ADMM) [35] strategy to accelerate convergence.

V subproblem:

Firstly, update V by fixing the other variables, and this subproblem can be expressed as

(20) $$\begin{array}{l} \underset{V}{\min}tr(V^{T}(FL{\hat{E}}^{T})V)\\ s.t.\;V^{T}V=I \end{array}$$

Denote $FL{\hat{E}}^{T}=Q$, then we can obtain

(21) $$\begin{array}{l} \underset{V}{\min}tr(V^{T}QV)\\ s.t.\;V^{T}V=I \end{array}$$

V is obtained using the above-shown eigenvalue decomposition problem whose optimal solution involves the set of *k* eigenvectors of the topmost *k* smallest eigenvalues of Q.

S subproblem:

(22) $$\underset{S}{\min}\lambda_{1}{\left\|S\right\|}_{*}+\frac{\mu }{2}{\left\|S-Z+\frac{Y_{3}}{\mu }\right\|}_{F}^{2}$$

Using the singular value thresholding (SVT), the optimal S is obtained as

(23) $$S=\Phi_{\frac{\lambda_{1}}{\mu }}(Z-\frac{Y_{3}}{\mu })$$

where Φ represents the soft-thresholding operator.

W subproblem:

(24) $$\begin{array}{l} \underset{W}{\min}\frac{1}{2}\sum_{i,j}{\left\|V^{T}e_{1,i}-V^{T}e_{1,j}\right\|}_{2}^{2}W_{ij}+\lambda_{3}{\left\|W\right\|}_{F}^{2}\\ s.t.\;W1=1,W_{ii}=0,W\geq 0 \end{array}$$

We can find that (24) is independent for each i, so $W_{i}$ can be solved separately as follows:

(25) $$\begin{array}{l} \underset{W_{i}}{\min}\frac{1}{2}\sum_{i,j}{\left\|V^{T}e_{1,i}-V^{T}e_{1,j}\right\|}_{2}^{2}W_{ij}+\lambda_{3}{\left\|W_{i}\right\|}_{F}^{2}\\ s.t.\;W1=1,W_{ii}=0,W\geq 0 \end{array}$$

E subproblem:

(26) $$\underset{E}{\min}\lambda_{2}{\left\|E\right\|}_{2,1}+\frac{\mu }{2}{\left\|H_{1}\circ X-H_{1}\circ Z-E+\frac{Y_{1}}{\mu }\right\|}_{F}^{2}+\frac{\mu }{2}{\left\|R-E+\frac{Y_{6}}{\mu }\right\|}_{F}^{2}$$

Denote $T=\frac{1}{2}(H_{1}\circ X-H_{1}\circ Z+R+\frac{Y_{1}}{\mu }+\frac{Y_{6}}{\mu })$, $\tau =\frac{\lambda_{2}}{\mu }$.

We can obtain the *i*-th column of the variable E as

(27) $$E(:,i)=\left\{ \begin{array}{l} \frac{\left\|t_{i}\right\|-\tau }{\left\|t_{i}\right\|}t_{i}\;,\;if\;\tau <\left\|t_{i}\right\|\\ 0\;,\;otherwise \end{array}\right.$$

$\hat{E}$ subproblem:

(28) $$\underset{\hat{E}}{\min}tr(V^{T}(FL{\hat{E}}^{T})V)+\mu {\left\|\hat{E}-E_{1}+\frac{Y_{4}}{\mu }\right\|}_{F}^{2}+\mu {\left\|F-\hat{E}+\frac{Y_{5}}{\mu }\right\|}_{F}^{2}$$

Set $\partial /\partial \hat{E}=0$, the optimized result can be obtained as

(29) $$\hat{E}=\frac{2\mu E_{1}-VV^{T}FL-2Y_{4}+2\mu F+2Y_{5}}{4\mu }$$

$E_{2}$ subproblem:

(30) $$\underset{E_{2}}{\min}{\left\|H_{2}\circ R-H_{2}\circ E_{1}-E_{2}+\frac{Y_{2}}{\mu }\right\|}_{F}^{2}$$

(31) $$E_{2}=(H_{2}\circ R)-(H_{2}\circ E_{1})+\frac{Y_{2}}{\mu }$$

F subproblem:

(32) $$\underset{F}{\min}tr(V^{T}(FL{\hat{E}}^{T})V)+\mu {\left\|F-\hat{E}+\frac{Y_{5}}{\mu }\right\|}_{F}^{2}$$

(33) $$F=\frac{2\mu E-VV^{T}\hat{E}L^{T}-2Y_{5}}{2\mu }$$

R subproblem:

(34) $$\underset{R}{\min}{\left\|H_{2}\circ R-H_{2}\circ E_{1}-E_{2}+\frac{Y_{2}}{\mu }\right\|}_{F}^{2}+{\left\|R-E+\frac{Y_{6}}{\mu }\right\|}_{F}^{2}$$

(35) $$R=(H_{2}\circ H_{2}\circ E_{1}+H_{2}\circ E_{2}-H_{2}\circ \frac{Y_{2}}{\mu }+E-\frac{Y_{6}}{\mu })÷(1+H_{2}\circ H_{2})$$

where ÷ represents element-wise division.

- Lagrange multipliers

In this section, we compute and update the Lagrange multipliers.

(36) $$Y_{1}=Y_{1}+\mu (H_{1}\circ X-H_{1}\circ Z-E)$$

(37) $$Y_{2}=Y_{2}+\mu (H_{2}\circ R-H_{2}\circ E_{1}-E_{2})$$

(38) $$Y_{3}=Y_{3}+\mu (S-Z)$$

(39) $$Y_{4}=Y_{4}+\mu (\hat{E}-E_{1})$$

(40) $$Y_{5}=Y_{5}+\mu (F-\hat{E})$$

(41) $$Y_{6}=Y_{6}+\mu (R-E)$$

## 4. Results

To better compare the denoising results of deep learning methods on remote sensing images, we selected the following classical deep learning algorithms as comparative methods: DnCNN [36], FFDNet [37], HSID-CNN [38], GRN [39], DD-CNN [40], and PDSNet [41].

In Section 4.1, we use the Mean Peak Signal-to-Noise Ratio (MPSNR) and Mean Structural Similarity Index (MSSIM) as evaluation metrics. In Section 4.3, we employ the Equivalent Number of Looks (ENL) and Edge Preservation Index (EPI) for evaluation. Our specific parameter settings are as follows: batch size of 150, ADAM optimizer, and learning rate of 5 × 10^−3^. For selecting parameters such as rank threshold, hierarchical levels, and regularization terms, we integrated our proposed HLR-DUR model into an AutoML tool. Across different datasets, we employed cross-validation to automatically search for optimal parameters.

### 4.1. Experiments on Optical Images

#### 4.1.1. Dataset Description

In this section, we selected the NWPU-RESISC45 dataset as the experimental subject. The NWPU-RESISC45 dataset is an optical remote sensing image dataset, which contains 45 different types of natural and man-made scenes, such as airports, bridges, forests, lakes, ports, etc., with approximately 700 different images for each type. These images are known for their high resolution and diversity, making them highly suitable for training and testing computer vision algorithms for image recognition, classification, and denoising. In order to conduct our experiments, the dataset was converted into single-channel grayscale images, and Gaussian noise with different variances of 0.01, 0.02, 0.04, 0.06, 0.08, and 0.1 was added to conduct the denoising experiments.

#### 4.1.2. Experiments and Analysis

**Figure 3:**
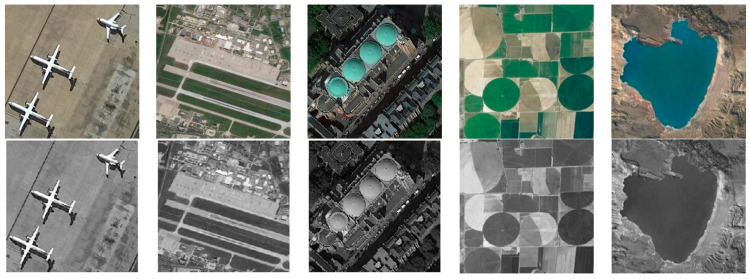
NWPU-RESISC45 dataset.

**Figure 4:**
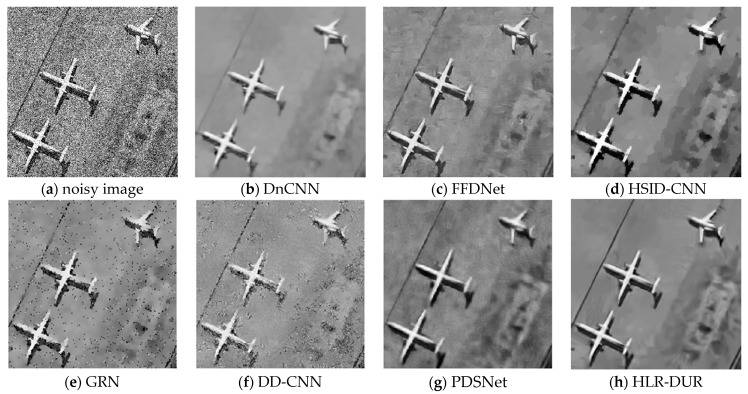
The denoising results of each comparative algorithm in Figure 3.

Noise with a variance of 0.04 is added to the first column of images in Figure 3 and Figure 4 show the denoising results for each comparison algorithm In Figure 4f, it is evident that DD-CNN exhibits the poorest visual performance, characterized by noticeable noise artifacts in the image and significant blur and deformation along the edges of the airplane. While DnCNN managed to recover the overall structure of the noise-free image, it suffered severe blurring in edge details. The lines in the top-left corner of the image were nearly entirely filtered out, and the airplane’s edges in the top-right corner appeared notably blurred. GRN retained some crucial edge information compared to the others but still exhibited noticeable noise residues, resulting in subpar visual quality. In contrast, FFDNet, HSID-CNN, PDSNet, and HLR-DUR achieved comparatively better denoising results, preserving more structural information in the image.

However, it can be seen that among all compared algorithms, HLR-DUR was the only method that clearly and completely preserved the lines in the upper left corner, indicating a significant advantage of HLR-DUR in edge preservation. Additionally, the shape and structure of the three airplanes in the denoising results of the HLR-DUR were closest to the original image. Although some texture information was missing in the denoised result of HLR-DUR, the overall edge structure was the most completely preserved. It is evident that introducing the hierarchical denoising model into the deep learning domain significantly improved the model’s edge preservation ability and denoising effect.

In this section, we evaluate the denoising results of the dataset under different noise levels using MPSNR and MSSIM as evaluation metrics. These metrics were chosen because they are commonly used to assess denoising performance across entire datasets, providing a comprehensive and reliable evaluation of algorithm performance. From the data in Table 1, it is evident that DnCNN, despite its poor visual effect in Figure 4, performed well on both MPSNR and MSSIM metrics, with its MPSNR results being second only to the HLR-DUR. GRN and DD-CNN achieved competitive denoising results at noise variances of 0.01, 0.02, and 0.04, but their MPSNR and MSSIM values dropped faster than other comparative algorithms when the noise pollution level became severe, indicating that these two algorithms may not effectively remove noise under high noise conditions. Meanwhile, FFDNet and HSID-CNN showed more stable performance but did not achieve the best results in numerical evaluation. HLR-DUR achieved the highest metric values in most cases, especially in PSNR. Even with a noise variance of 0.1, the MPSNR value obtained by HLR-DUR remained higher than 30, showing a clear advantage over other algorithms. As for the MSSIM metric, HLR-DUR achieved the best values under different levels of noise, indicating HLR-DUR’s superiority in structure preservation. 

### 4.2. Experiments on Hyperspectral Images

#### 4.2.1. Dataset Description

**Figure 5:**
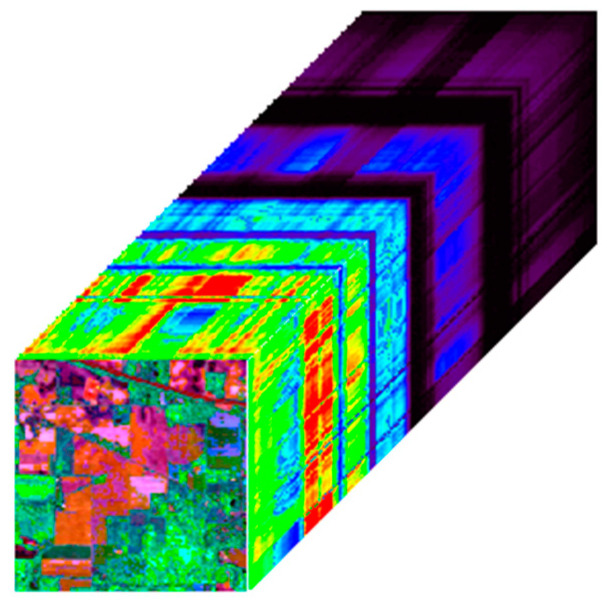
AVIRIS Indian Pines dataset (R: 29, G: 42, B: 89).

The AVIRIS Indian Pines dataset was acquired in 1992 by the Airborne Visible/Infrared Imaging Spectrometer (AVIRIS) sensor over the Indian Pines test site in northwestern Indiana. The data size is 145 × 145 × 220, and the pseudo-color image is shown in Figure 5. Several bands in this dataset contain mixed noise, including impulse noise and Gaussian noise.

#### 4.2.2. Experiments and Analysis

**Figure 6:**
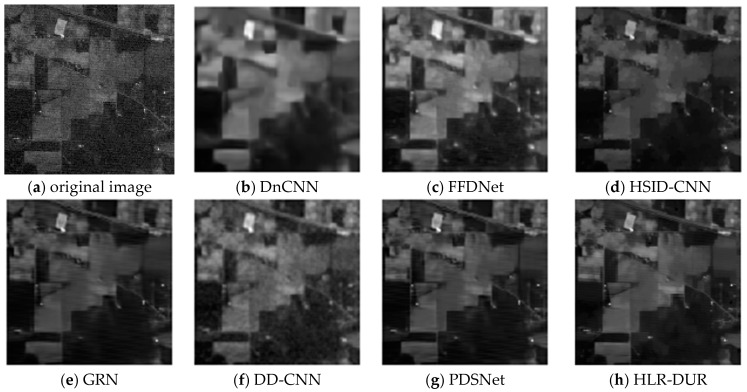
The denoising results of all algorithms on the Pavia city center dataset.

Firstly, Figure 6 shows the denoising results of all algorithms on the Pavia city center dataset. FFDNet, HSID-CNN, and GRN all achieved good denoising results for hyperspectral images, while DnCNN caused obvious edge blurring and DD-CNN failed to effectively remove noise. Furthermore, both PDSNet and our proposed HLR-DUR model excelled in removing noise from homogeneous regions while preserving edge information, achieving superior visual outcomes.

**Figure 7:**
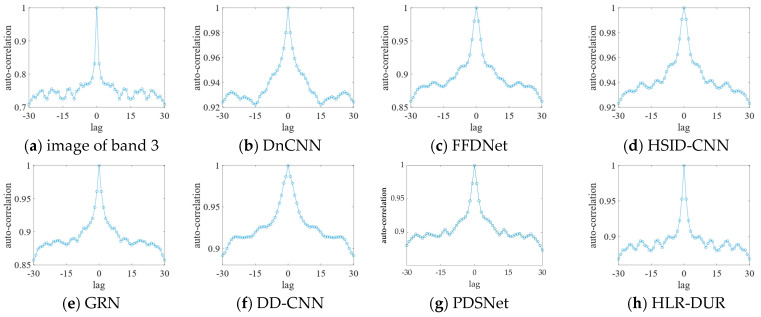
The auto-correlation curves of the denoised images.

Figure 7 shows the auto-correlation curves of the denoised images. The auto-correlation curve depicts the correlation of the image at different lags, reflecting the consistency and repetitive pattern of the image texture. The closer the autocorrelation curve is to that of the original image, the better the denoising algorithm performs in preserving the image texture and structure. This proximity indicates that the algorithm can effectively remove noise while preserving edge details and texture features, which is crucial for maintaining image quality. Although the HLR-DUR model and the PDSNet model showed comparable visual effects, the results in Figure 6 clearly demonstrate that our proposed HLR-DUR model better preserves the details of the original image, as evidenced by its autocorrelation curve closely resembling that of the original image. Other algorithms showed a certain degree of broadening, which is typically the result of over-smoothing. Over-smoothing leads to the loss of unique texture details in the image, creating an unnatural visual effect, which is undesirable in image processing. The broadening observed in the comparative algorithms indicates that while noise was removed, a significant amount of texture information was also discarded. In summary, the analysis of the auto-correlation curves revealed that HLR-DUR exhibited superior ability in preserving the original texture and structure of the image.

### 4.3. Experiments on SAR Images

#### 4.3.1. Dataset Description

**Figure 8:**
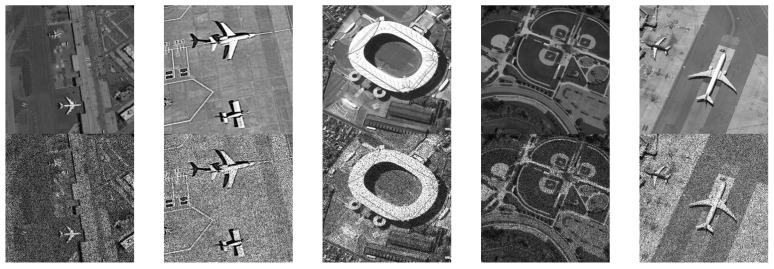
The samples of the Virtual SAR dataset.

In the experiments of this section, we used the Virtual SAR dataset as the experimental subject. The Virtual SAR dataset is a simulated SAR image dataset, which randomly adds different levels of speckle noise based on the NWPU-RESISC45 dataset. The Virtual SAR dataset consists of 31,500 images, each with a size of 256 × 256. Each image has a corresponding noise-free version and a simulated noise-added version, as shown in Figure 8.

#### 4.3.2. Experiments and Analysis

**Figure 9:**
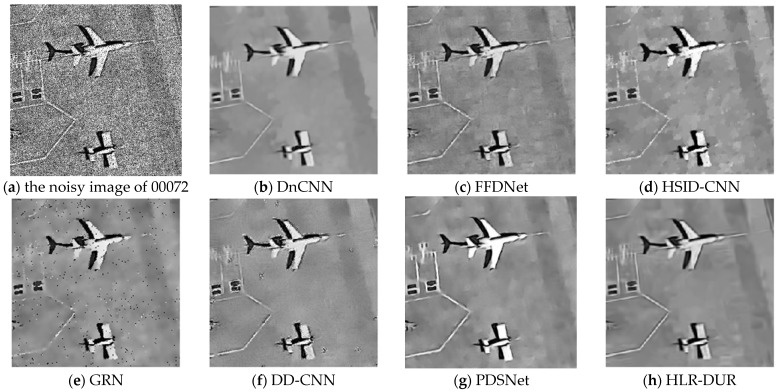
Denoised results of the Virtual SAR dataset.

As shown in Figure 9, taking data number 00072 from the Virtual SAR dataset as an example, the denoising results of various comparative algorithms are presented. Among all the methods, the denoising result obtained by the GRN algorithm still had obvious noise affecting the image quality, while the FFDNet and HSID-CNN algorithms failed to smoothly denoise the homogeneous areas, resulting in some noisy spots in the image. DnCNN, DD-CNN, and PDSNet, which are better at smoothing noise, did not adequately preserve edge information, with the runway lines in the upper left corner of these two images almost completely filtered out. In other algorithms, these lines were partially or completely preserved. Visually, HLR-DUR still achieved the best effect. It is observed that the structural outline of the airplane was clearer in the result of HLR-DUR, and the denoising effect in the homogeneous areas was also better.

**Figure 10:**
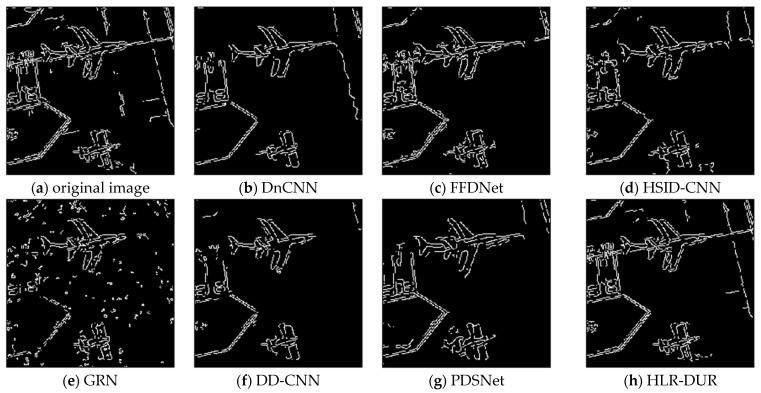
Comparison of the edge extraction results after denoising by comparative algorithms.

To further evaluate the edge preservation capabilities of different denoising algorithms on SAR images, we used the Canny edge detection operator to extract edges from the denoised results of each comparative algorithm, as shown in Figure 10. The Canny edge detection operator is widely used in various image processing and computer vision tasks, including image segmentation, feature extraction, and pattern recognition. Although it is not the most advanced algorithm, due to its reliability, it remains one of the most popular edge detection methods in image processing. In the extracted edge results, it can be seen that due to incomplete noise removal in GRN, its edge extraction was also affected. While other comparative algorithms generally extracted clear and complete target edges, specifically the edge extracted by HLR-DUR was the most complete and closest to the original image edges.

Table 2 shows the numerical evaluation of the denoising performance of various comparative algorithms on five images from the Virtual SAR dataset. Because we were evaluating the denoising performance of SAR images, we employed ENL and EPI as evaluation metrics to assess the despeckling and edge preservation capabilities. It can be seen that on the SAR dataset, the HLR-DUR model performed well on both ENL and EPI metrics, meaning it effectively maintains the image’s edges and details while reducing noise. Other algorithms such as DnCNN and DD-CNN exhibited poorer performance in edge preservation compared to FFDNet, HSID-CNN, GRN, and PSDNet. While FFDNet, HSID-CNN, GRN, and PSDNet also achieved respectable results in terms of ENL and EPI, overall, there remains a gap compared to HLR-DUR.

**Figure 11:**
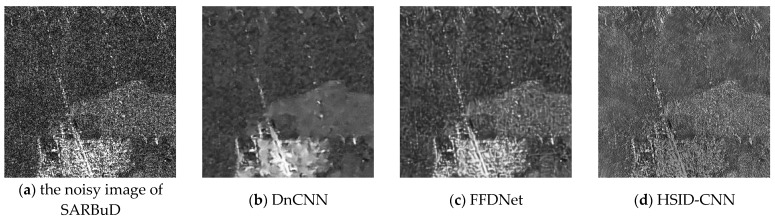

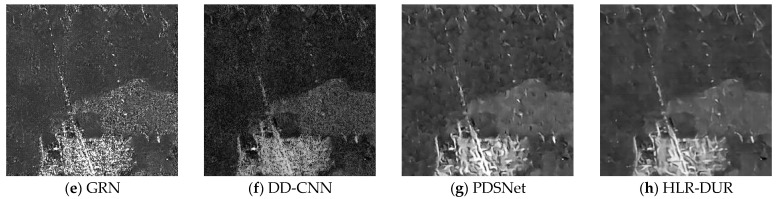
Denoised results of the SARBuD dataset.

In Figure 11, we present the denoising results obtained from a real SAR dataset, SARBuD. The SARBuD dataset is a synthetic aperture radar dataset designed for benchmarking despeckling algorithms. It includes a variety of simulated SAR images with different noise levels, providing a comprehensive resource for evaluating the performance of image-denoising techniques. It can be observed that FFDNet, HSID-CNN, and GRN algorithms exhibit suboptimal performance with residual noise remaining in uniform regions. While DnCNN, DD-CNN, and PDSNet demonstrate relatively effective denoising, they tend to blur or even filter out image edges. HLR-DUR achieved the best visual results among the methods evaluated. 

As shown in Table 3, it is evident that the HLR-DUR model consistently outperformed other algorithms when faced with real SAR image datasets. This indicates that it achieves superior evaluation metrics in most scenarios, suggesting its capability to effectively balance denoising and edge preservation in real SAR images.

The HLR-DUR model showed significant advantages through many evaluation metrics. By conducting a comprehensive analysis of the denoising results for virtual SAR images and real SAR images, it can be seen that compared to other commonly used denoising algorithms such as DnCNN, FFDNet, HSID-CNN, GRN, DD-CNN, and PDSNet, HLR-DUR more effectively preserves the image’s edge parts and detail information while reducing noise levels. Especially in dealing with complex and diverse image denoising problems, HLR-DUR demonstrated its comprehensive advantages, achieving the goal of edge preservation and denoising, and holds great potential in the field of image denoising.

### 4.4. Processing Speed and Ablation Experiments

#### 4.4.1. Ablation Experiment and Accuracy and Loss Curves

**Figure 12:**
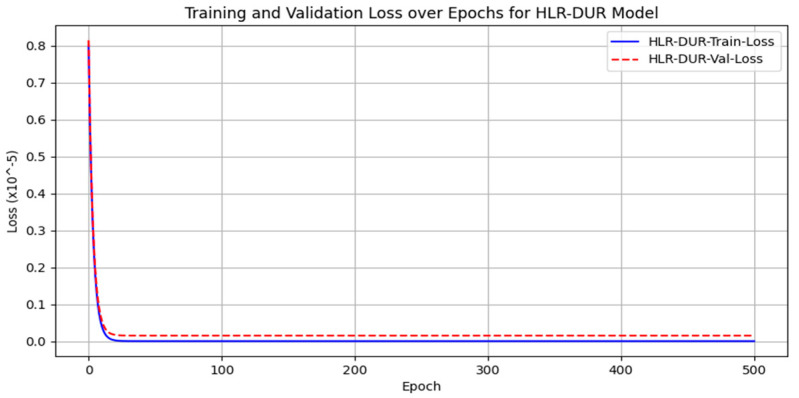
Training accuracy and loss curves.

Figure 12 illustrates the training and validation loss results of HLR-DUR, demonstrating its rapid convergence and minimal loss values, indicating effective denoising performance.

We conducted ablation experiments on the deep model. The primary objective of these experiments was to assess the impact of the edge modules incorporated within the model. The results presented in Table 4 unequivocally demonstrate that integrating the edge module significantly improved both the denoising efficacy and the model’s ability to preserve edges. This outcome robustly validates the effectiveness of the edge module in enhancing overall model performance.

#### 4.4.2. Results of the Processing Speed and Parameters Experiment

We employed four NVIDIA^®^ GeForce RTX 3090 GPUs which are manufactured by NVIDIA Corporation, a technology company based in Santa Clara, California, United States.to evaluate the processing speed and parameter count of training models using 256×256 SAR images as input. Table 5 presents the parameter sizes and processing details of seven models. Among them, the model with the largest parameter count was the enhanced DD-CNN, which totaled 7,955,552 parameters. The model requiring the longest computational time was PDSNet, with an approximate execution time of 76.26 ms. Our proposed HLR-DUR model had 28,311 parameters and took about 1.2 ms per epoch to compute.

## 5. Conclusions

In this paper, a new deep low-rank model for remote sensing image denoising named HLR-DUR was proposed. Compared with existing denoising methods, HLR-DUR combines the advantages of the traditional LRR model and deep learning, enabling it to extract edge information using prior knowledge regulations while further enhancing the model’s denoising performance and computational efficiency through the deep autoencoder. HLR-DUR combines the shallow low-rank denoising model with an autoencoder to construct a two-level deep encoder. It uses deep unfolding to extract both the low-rank and edge components. By integrating traditional iterative algorithms with the deep learning framework, the iterative steps are unfolded into multiple layers, which are then aggregated to obtain the final denoised result. Experiments conducted on three types of remote sensing datasets, optical remote sensing images, hyperspectral images, and SAR images, demonstrated that HLR-DUR achieves better edge-preserving denoising results than SOTA models and significantly improves the denoising effect.

## Figures and Tables

**Table 1:** Evaluation of denoising results on the NWPU-RESISC45 dataset.

Noise Variance	Evaluation Metrics	DnCNN	FFDNet	HSID-CNN	GRN	DD-CNN	PDSNet	HLR-DUR
0.01	MPSNR	33.8562	29.1558	25.1696	23.4655	24.7268	28.4567	36.8927
	MSSIM	0.7627	0.7924	0.7248	0.7085	0.7376	0.7073	0.7706
0.02	MPSNR	31.9557	27.5778	25.0029	22.9981	22.9527	25.4539	36.4328
	MSSIM	0.7250	0.7176	0.7222	0.6749	0.6778	0.7018	0.7625
0.04	MPSNR	29.7556	26.0261	24.4276	21.1774	19.5923	23.5846	35.6956
	MSSIM	0.6772	0.6445	0.7106	0.4552	0.3993	0.6936	0.7433
0.06	MPSNR	28.3637	25.1928	23.8439	17.4169	16.8556	20.3977	34.9296
	MSSIM	0.6562	0.5838	0.6651	0.2329	0.1986	0.5725	0.6706
0.08	MPSNR	27.7164	24.1979	23.1341	17.3257	14.8928	18.2633	32.9710
	MSSIM	0.6366	0.5186	0.5973	0.2312	0.1250	0.5591	0.6290
0.1	MPSNR	27.0592	23.4049	22.3332	14.5862	14.2963	18.0361	30.8986
	MSSIM	0.5575	0.4681	0.5214	0.1491	0.1099	0.4826	0.5928

**Table 2:** Evaluation of denoising results on the Virtual SAR dataset.

		Noisy Image	DnCNN	FFDNet	HSID- CNN	GRN	DD-CNN	PDSNet	HLR-DUR
Image 1	ENL	5.5820	103.5885	118.0177	114.4488	125.6164	98.0918	116.3475	125.9335
	EPI	-	0.5839	0.6674	0.6937	0.7022	0.5803	0.6309	0.7356
Image 2	ENL	9.3213	96.2285	95.7900	101.6122	104.6173	84.0133	107.3329	115.3813
	EPI	-	0.5236	0.5937	0.6796	0.6725	0.6116	0.5682	0.6823
Image 3	ENL	7.6195	107.8969	116.3891	113.5986	116.4556	100.3012	114.2744	124.5229
	EPI	-	0.6365	0.8779	0.8727	0.8991	0.6013	0.8917	0.9164
Image 4	ENL	7.7927	76.6310	73.9612	80.8328	80.9381	60.4646	83.6534	81.9254
	EPI	-	0.5929	0.7398	0.8634	0.8929	0.7013	0.7972	0.9147
Image 5	ENL	8.8592	103.7392	109.3430	116.1278	110.9981	101.4649	117.5947	125.6757
	EPI	-	0.7030	0.8562	0.8519	0.8244	0.7778	0.8139	0.8662

**Table 3:** Evaluation of denoising results on the SARBuD dataset.

		Noisy Image	DnCNN	FFDNet	HSID- CNN	GRN	DD-CNN	PDSNet	HLR-DUR
Image 1	ENL	10.7727	67.4415	55.9390	54.1302	57.7159	58.7127	65.9892	72.2878
	EPI	-	0.4201	0.5413	0.5265	0.5159	0.5337	0.5712	0.6175
Image 2	ENL	12.6964	62.5110	67.8803	63. 4276	63.5245	69.7125	77. 0636	92.3050
	EPI	-	0.6521	0.7019	0.7060	0.8226	0.6715	0.8064	0.8626
Image 3	ENL	10.6408	56.4970	63.1859	70.1377	66.9889	54.0735	82.4010	78.4357
	EPI	-	0.6361	0.71845	0.6423	0.7223	0.6531	0.7312	0.7907

**Table 4:** Results of the ablation experiments.

	Evaluation Metrics	0.01	0.02	0.04	0.06	0.08	0.1
Without Edge Module	MPSNR	35.3927	35.0233	34.6201	33.2930	31.9223	29.1146
	MSSIM	0.6111	0.6032	0.5725	0.5549	0.7022	0.5803
HLR-DUR	MPNSR	36.8927	36.4328	35.6956	34.9296	32.9710	30.8986
	MSSIM	0.7023	0.6882	0.6620	0.6321	0.6290	0.6116

**Table 5:** Different model training parameters and time consumption comparison.

Methods	DnCNN	FFDNet	HSID- CNN	GRN	DD-CNN	PDSNet	HLR-DUR
Time(ms)	22.51	50.13	47.13	67.78	63.16	76.26	1.2
Parameters	667,008	485,316	556,097	1,322,251	7,955,552	1,366,111	28,311

## Data Availability

The original data presented in the study are openly available in [IEEEDataport and Puedue University Research Repository] at [https://ieee-dataport.org/open-access/virtual-sar-synthetic-dataset-deep-learning-based-speckle-noise-reduction-algorithms and https://purr.purdue.edu/publications/1947/1].

## References

[1] Sabins F.F., Ellis J.M. (2020). Remote Sensing: Principles, Interpretation, and Applications.

[2] Rasti B., Chang Y., Dalsasso E., Denis L., Ghamisi P. (2021). Image restoration for remote sensing: Overview and toolbox. IEEE Geosci. Remote Sens. Mag..

[3] Li G., Gu X., Chen C., Zhou C., Xiao D., Wan W., Cai H. (2024). Low-Frequency Magnetotelluric Data Denoising Using Improved Denoising Convolutional Neural Network and Gated Recurrent Unit. IEEE Trans. Geosci. Remote Sens..

[4] Li G., Wu S., Cai H., He Z., Liu X., Zhou C., Tang J. (2023). IncepTCN: A new deep temporal convolutional network combined with dictionary learning for strong cultural noise elimination of controlled-source electromagnetic data. Geophysics.

[5] Han L., Zhao Y., Lv H., Zhang Y., Liu H., Bi G. (2022). Remote sensing image denoising based on deep and shallow feature fusion and attention mechanism. Remote Sens..

[6] Huang Z., Zhu Z., Wang Z., Shi Y., Fang H., Zhang Y. (2023). DGDNet: Deep gradient descent network for remotely sensed image denoising. IEEE Geosci. Remote Sens. Lett..

[7] Tian C., Fei L., Zheng W., Xu Y., Zuo W., Lin C.-W. (2020). Deep learning on image denoising: An overview. Neural Netw..

[8] Jiao L., Zhao J. (2019). A survey on the new generation of deep learning in image processing. IEEE Access.

[9] Feng X., Zhang W., Su X., Xu Z. (2021). Optical remote sensing image denoising and super-resolution reconstructing using optimized generative network in wavelet transform domain. Remote Sens..

[10] Huang Z., Zhang Y., Li Q., Li Z., Zhang T., Sang N., Xiong S. (2019). Unidirectional variation and deep CNN denoiser priors for simultaneously destriping and denoising optical remote sensing images. Int. J. Remote Sens..

[11] Xie Y., Qu Y., Tao D., Wu W., Yuan Q., Zhang W. (2016). Hyperspectral image restoration via iteratively regularized weighted schatten $ p $-norm minimization. IEEE Trans. Geosci. Remote Sens..

[12] Dong W., Wang H., Wu F., Shi G., Li X. (2019). Deep spatial–spectral representation learning for hyperspectral image denoising. IEEE Trans. Comput. Imaging.

[13] Gu F., Zhang H., Wang C. (2020). A two-component deep learning network for SAR image denoising. IEEE Access.

[14] Feng X., Tian S., Abhadiomhen S.E., Xu Z., Shen X., Wang J., Zhang X., Gao W., Zhang H., Wang C. (2023). Edge-Preserved Low-Rank Representation via Multi-Level Knowledge Incorporation for Remote Sensing Image Denoising. Remote Sens..

[15] Chen Y., Huang T.-Z., He W., Zhao X.-L., Zhang H., Zeng J. (2021). Hyperspectral image denoising using factor group sparsity-regularized nonconvex low-rank approximation. IEEE Trans. Geosci. Remote Sens..

[16] He W., Zhang H., Zhang L., Shen H. (2015). Hyperspectral image denoising via noise-adjusted iterative low-rank matrix approximation. IEEE J. Sel. Top. Appl. Earth Obs. Remote Sens..

[17] Ma T.-H., Xu Z., Meng D. (2020). Remote sensing image denoising via low-rank tensor approximation and robust noise modeling. Remote Sens..

[18] Zhang H., Liu L., He W., Zhang L. (2019). Hyperspectral image denoising with total variation regularization and nonlocal low-rank tensor decomposition. IEEE Trans. Geosci. Remote Sens..

[19] Li C., Ma Y., Huang J., Mei X., Ma J. (2015). Hyperspectral image denoising using the robust low-rank tensor recovery. J. Opt. Soc. Am. A.

[20] Guan D., Xiang D., Tang X., Kuang G. (2019). SAR image despeckling based on nonlocal low-rank regularization. IEEE Trans. Geosci. Remote Sens..

[21] Chen G., Li G., Liu Y., Zhang X.-P., Zhang L. (2020). SAR image despeckling based on combination of fractional-order total variation and nonlocal low rank regularization. IEEE Trans. Geosci. Remote Sens..

[22] Geng J., Fan J., Ma X., Wang H., Cao K. An iterative low-rank representation for SAR image despeckling. Proceedings of the 2016 IEEE International Geoscience and Remote Sensing Symposium (IGARSS).

[23] Baier G., He W., Yokoya N. (2020). Robust nonlocal low-rank SAR time series despeckling considering speckle correlation by total variation regularization. IEEE Trans. Geosci. Remote Sens..

[24] Zhang H., Chen H., Yang G., Zhang L. (2021). LR-Net: Low-rank spatial-spectral network for hyperspectral image denoising. IEEE Trans. Image Process..

[25] Nguyen H.V., Ulfarsson M.O., Sigurdsson J., Sveinsson J.R. Deep sparse and low-rank prior for hyperspectral image denoising. Proceedings of the IGARSS 2022–2022 IEEE International Geoscience and Remote Sensing Symposium.

[26] Sun H., Liu M., Zheng K., Yang D., Li J., Gao L. (2021). Hyperspectral image denoising via low-rank representation and CNN denoiser. IEEE J. Sel. Top. Appl. Earth Obs. Remote Sens..

[27] Zhang Q., Yuan Q., Song M., Yu H., Zhang L. (2022). Cooperated spectral low-rankness prior and deep spatial prior for HSI unsupervised denoising. IEEE Trans. Image Process..

[28] Xiong K., Zhao G., Wang Y., Shi G. (2021). SAR imaging and despeckling based on sparse, low-rank, and deep CNN priors. IEEE Geosci. Remote Sens. Lett..

[29] Monga V., Li Y., Eldar Y.C. (2021). Algorithm unrolling: Interpretable, efficient deep learning for signal and image processing. IEEE Signal Process. Mag..

[30] Passah A., Amitab K., Kandar D. (2021). SAR image despeckling using deep CNN. IET Image Process..

[31] Wang S., Wang X., Zhang L., Zhong Y. (2022). Deep low-rank prior for hyperspectral anomaly detection. IEEE Trans. Geosci. Remote Sens..

[32] Candès E.J., Li X., Ma Y., Wright J. (2011). Robust principal component analysis?. J. ACM.

[33] Dong Y., Chan R.H., Xu S. (2007). A detection statistic for random-valued impulse noise. IEEE Trans. Image Process..

[34] Lopes A., Touzi R., Nezry E. (1990). Adaptive speckle filters and scene heterogeneity. IEEE Trans. Geosci. Remote Sens..

[35] Hong M., Luo Z.-Q. (2017). On the linear convergence of the alternating direction method of multipliers. Math. Program..

[36] Huang Z., Wang Z., Zhu Z., Zhang Y., Fang H., Shi Y., Zhang T. (2022). DLRP: Learning deep low-rank prior for remotely sensed image denoising. IEEE Geosci. Remote Sens. Lett..

[37] Zhang K., Zuo W., Zhang L. (2018). FFDNet: Toward a fast and flexible solution for CNN-based image denoising. IEEE Trans. Image Process..

[38] Yuan Q., Zhang Q., Li J., Shen H., Zhang L. (2018). Hyperspectral image denoising employing a spatial–spectral deep residual convolutional neural network. IEEE Trans. Geosci. Remote Sens..

[39] Cao X., Fu X., Xu C., Meng D. (2021). Deep spatial-spectral global reasoning network for hyperspectral image denoising. IEEE Trans. Geosci. Remote Sens..

[40] Shan W., Liu P., Mu L., Cao C., He G. (2019). Hyperspectral image denoising with dual deep CNN. IEEE Access.

[41] Lin C., Qiu C., Jiang H., Zou L. (2023). A Deep Neural Network Based on Prior-Driven and Structural Preserving for SAR Image Despeckling. IEEE J. Sel. Top. Appl. Earth Obs. Remote Sens..

